# Assessment of the steps taken towards avoidance of medication errors among hypertensive outpatients attending a tertiary health care facility in Nigeria: a cross-sectional study

**DOI:** 10.11604/pamj.2019.33.76.13594

**Published:** 2019-06-03

**Authors:** Kosisochi Chinwendu Amorha, Glory James, Chibueze Anosike, Mathew Jegbefume Okonta

**Affiliations:** 1 Department of Clinical Pharmacy and Pharmacy Management, Faculty of Pharmaceutical Sciences, University of Nigeria, Nsukka, Enugu State, Nigeria

**Keywords:** Clinic visits, hypertension, medication errors

## Abstract

**Introduction:**

Active involvement of patients in the management of their health has been suggested as a major means of curtailing medication errors. This study aimed to assess the steps taken by hypertensive patients in avoiding medication errors before, during and after clinic visits

**Methods:**

A cross-sectional study was conducted in Enugu State University Teaching Hospital (ESUTH), Parklane, Enugu, Nigeria (June to August, 2016) using a standardized 35-item interviewer-administered questionnaire. The IBM SPSS Version 20.0 was utilized for statistical analysis with P < 0.05, considered statistically significant

**Results:**

A total of 200 questionnaires were completed and returned. Few (24.4%) of the respondents were taking non-prescribed medicines and most (61.5%) knew their names. Only 41.9% of the patients monitor their blood pressure very often. There was a statistical difference between the mean scores of steps to avoid medication errors after the clinic visit for the different occupations (F = 8.109; P < 0.001) and educational level (F = 6.182; P < 0.001)

**Conclusion:**

Patients that took necessary steps in avoiding medication errors before their clinic visits were likely to avoid errors at the clinic. Also, patients that avoided medication errors at the clinic were likely to avoid medication errors after the doctor's visit.

## Introduction

Hypertension, a major public health problem, has been identified as a leading cause of morbidity and mortality worldwide [1, 2]. Uncontrolled hypertension is the most common risk factor for the development of stroke, heart failure (HF), chronic kidney disease and coronary artery disease in sub-Saharan Africa [3, 4]. However, these complications can be prevented by ensuring adequate blood pressure control through accurate and consistent use of drugs and non-drug therapy, as recommended by the healthcare professionals [5]. Medication errors in hypertensive patients may arise in the course of prescribing, dispensing or administration of drugs [6]. Admittedly, the provision of drug therapy by a healthcare professional to a patient is a complex process. Thus, medication error may occur at any step along the way, from prescribing to the ultimate provision of the drug to the patient [7]. In view of this, the National Coordinating Council for Medication Error and Prevention (NCCMERP) described medication errors as, “any preventable event that may cause or lead to inappropriate medication use or patient harm, while the medication is in the control of the health care professional, patient, or consumer. Such events may be related to professional practice, health care products, procedures and systems including: prescribing; order communication; product labeling, packaging and nomenclature, compounding, dispensing, distribution, administration, education, monitoring and use” [8, 9]. Unfortunately, medication errors account for up to a third of all medical errors in the hospital and may lead to adverse outcomes such as increased mortality rates, increased length of hospital stay and high medical expenses [10, 11]. The responsibility for the prevention of medication errors involves not just the healthcare professionals and healthcare systems but also the patients themselves. To help curtail this incidence, patients are now being actively engaged in the management of their medications [12, 13]. Thus, patients should not just be informed of the names of their medications but also the reasons for their use, time of administration, how much and how often to take it, duration of therapy, common side effects that could occur, what to do about missed doses, common interactions with other drugs or foods, and the correct dose. Hence, patients are well prepared to act as the final check in the system. When patients take an active and informed role in their healthcare, many errors can be prevented [14-16]. Against this backdrop, the general objective of this study was to assess the steps taken among hypertensive outpatients towards avoiding medication errors before, during and after clinic visits.

## Methods

Medication errors are preventable events that may cause or lead to inappropriate medication use or patient harm, while the medication is in the control of the health care professional, patient, or consumer [8, 9]. The steps taken by the hypertensive patients to reduce medication errors before consulting with their doctors include knowing their last clinic visit, the complaints that brought them to the hospital, the name and phone number of the doctor they consulted, the number and names of the non-prescription drugs they are on. The steps taken by the hypertensive patients to reduce medication errors at the consultation room include knowing their diagnosis, requesting for the names of their medications, how and when to take their prescribed medicines, possible side effects and what to do if they occur, activities to avoid while taking their prescribed medicines, possible interactions with food, other drugs or alcohol, special storage conditions. The steps taken by hypertensive patients to reduce medication errors after consulting with their doctors include calling their doctor/pharmacist when they have problems with their medications such as troublesome side effects or to seek information on their prescribed medications and knowing the date of their next clinic visit.

**Study design and sample population:** this was a cross-sectional study conducted in Enugu State University Teaching Hospital (ESUTH), Parklane, Enugu, Nigeria. The study participants included registered hypertensive patients in the Cardiology Clinic of the hospital who gave their consent to participate. Critically-ill patients were exempted from the study. A sample size of 281 out of a population of 1,040 hypertensive patients that visited the cardiology unit within the stipulated time frame of 1 month was obtained using the Raosoft^®^ sample size calculator.

**Data collection:** data were collected using a 35-item structured, interviewer-administered questionnaire. To ensure quality of the data, questions were adapted from a previously conducted study by the National Council on Patient Information and Education [17] and changes made based on local context. The questionnaire was content validated by clinical pharmacy lecturers in the University of Nigeria, Nsukka and pre-tested among ten hypertensive patients in ESUTH who were not included in the study. This was to eliminate ambiguities. Beside the demographics, the questionnaire had sections to assess the steps taken by hypertensive patients in avoiding medication errors before, during and after clinic visits. The sections for during and after clinic visits were filled after the patients had seen the doctor. The study respondents were given the questionnaires to complete during their cardiology clinic visits as they waited to see a physician and after they had seen the physician. The selected participants completed the questionnaires in about 15 minutes without seeking help from other patients or healthcare professionals. Data were collected between June to August, 2016.

**Data analysis:** data obtained were entered and analyzed using the IBM SPSS Version 20.0. Descriptive statistics were used to analyze the steps taken among respondents to avoid medication errors. Analysis of variance (ANOVA) was utilized to determine the difference between the demographic variables in their mean steps to avoid medication errors. The Pearson Chi-square test and Pearson correlation test were used, where applicable, to show the association and relationship between variables. For all results, P < 0.05 was considered statistically significant.

**Ethical consideration:** ethical clearance was obtained from the Health Research and Ethics Board of the Enugu State University Teaching Hospital (ESUTH), Parklane, Enugu, prior to the commencement of this study. The study was conducted based on the approved protocol. The participants were informed of the objectives of the study and their confidentiality was fully maintained.

## Results

**Socio-demographic information:** the socio-demographic characteristics of the 281 patients assessed in the study, showed that majority of the respondents (54.8%) were greater than 60 years of age and most (80.3%) were married. In addition, occupational status of the participants indicated that few were unemployed (22.9%) and had primary education (31.4%) (Table 1). Before consultation with their doctor, most (82.7%) of the patients remembered the date of their last clinic visit, although many (86.9%) did not have their doctor's phone number. Additionally, 41.9% of the patients claimed to often monitor their blood pressure (Table 2). During consultation with their doctor, few patients (11.1%) were given their doctors' phone number upon referral and about 44.9% knew the name of their prescribed drugs. Most of the patients (89.7%) were informed on how to take their drugs. About a fifth indicated that they were informed about the possible side effects of their medications with a similar percentage admitting that they were told what to do when side effects occur. Most of the patients agreed that it was not safe to take their drugs together with alcohol. Less than half of them admitted that they were informed on the foods and medications to avoid, activities to avoid while on drugs, and how to know if their drug is working. After consultation with their doctor, few of the patients (19.8%) contact their doctor or pharmacist when they experience troublesome side effects. However, most of the patients knew the date of their next clinic visit (Table 3). The mean difference analysis using ANOVA showed a statistically significant difference between the mean scores on steps to avoid medication errors before consultation with doctors (P = 0.033, F = 2.985). There was also a significant difference in the respondents' occupational status and the mean scores of steps to avoid medication errors at the consultation room (P = 0.034, F = 2.661). Those in civil service took the best steps in avoiding medication errors at the consultation room. Also, there was a statistically significant difference in the patients' mean scores of steps to avoid medication errors before consultation with the doctor (P = 0.001, F = 6.772) at the consultation room (P = 0.001, F = 6.182) and after consulting with the doctor (P = 0.001, F = 7.983) for the different educational levels. Those who had tertiary education took the best steps in avoiding medication errors before consultation with their doctor (P = 0.001), in the consultation room with the doctor (P = 0.001) and after consultation (P = 0.018) (Table 4). There were associations between patients' educational status and the steps taken to avoid medication errors before consultation (χ^2^ = 13.164, P < 0.004), at the consultation room (χ^2^ = 38.404, P < 0.005) and after consultation (χ^2^ = 25.555, P < 0.001); with patients who had tertiary education taking the best steps to avoid medication errors at the different times (Table 5). There was an association between occupational status and steps taken to avoid medication errors at the consultation room (χ^2^ = 23.992, P < 0.001) and after consultation with the doctor (χ^2^ = 28.226, P < 0.005). Patients who were government-employed took the best steps towards avoiding medication errors during and after consultations with their doctors (Table 6).

**Table 1:** Socio-demographic information

Variables	n (%)
Age (in years)	
˂ 30	13(6.5)
30-60	77(38.7)
˃ 60	109(54.8)
**Marital Status**	
Single	12(6.4)
Married	151(80.3)
Divorced	19(10.1)
Widowed	6(3.2)
O**ccupation**	
Government employed	36(19.1)
Corporate employed	34(18.1)
Self-employed	32(17.0)
Unemployed	43(22.9)
E**ducational Status**	
Primary school	61(31.4)
Secondary school	45(23.2)
Tertiary School	57(29.4)
None	31(16.0)

**Table 2:** Hypertensive patients’ responses before consultation with doctor

VARIABLE	n (%)
**Do you know the date of your last clinic visit?**	
Yes	163(82.7)
No	34(17.3)
**Do you know your doctor’s name?**	
Yes	124(62.3)
No	75(37.7)
**Do you have your doctor’s phone number?**	
Yes	26(13.1)
No	172(86.9)
**What was the reason for your last clinic visit?**	
Appointment	35(17.9)
Check up	117(57.4)
Elevated BP	37(19.0)
Referrals	4(2.1)
Diabetes mellitus	1(0.5)
Arthritic pain	2(1.0)
Heart problems	3(1.5)
**How long did you have the problem?**	
Few hours	8(5.0)
Few days	16(10.0)
More than one week	99(62.3)
No problem	36(22.6)
**How many non-prescribed drugs are you on?**	
1	27(16.9)
2	8(5.0)
3	3(1.9)
**Not on any non-prescribed drugs**	121(75.6)
Many (˃3)	1(0.6)
Do you know their names	
Yes	24(15.0)
No	15(9.4)
Not on any non-prescribed drugs	121(75.6)
**Can you write down their names?**	
Aceclofenac	14(7.0)
Diclofenac	11(5.5)
None	120(60)
Paracetamol	1(0.5)
Yeast	4(2.0)
Ibuprofen	2(1.0)
No	8(4.0)
**How often do you monitor your blood pressure?**	
Very often	80(41.9)
Sometimes	83(43.5)
Rarely	28(14.9)

**Table 3:** Hypertensive patients’ responses after consultation with doctor

VARIABLE	n (%)
**IN THE CONSULTATION ROOM**	
**Did your doctor tell you the name of your medical problem?**	
Yes	149(74.9)
No	50(25.1)
**When referred, were you given the doctor’s phone number?**	
Yes	17(11.1)
No	136(88.9)
**Do you know the name of your medicine(s) prescribed**	
Yes	89(44.9)
No	109(55.1)
**Were you told how to take it/them**	
Yes	175(89.7)
No	20(10.3)
**Were you told when to take it**	
Yes	161(82.6)
No	23(17.4)
**Were you told the possible side effect(s) of your prescribed medicine**	
Yes	39(20.1)
No	155(79.9)
**Were you told what to do when the side effect(s) occur**	
Yes	37(23.9)
No	118(76.1)
**Were you told to take it with/without food**	
Yes	116(59.2)
No	80(40.4)
**Is it safe to take alcohol with your prescribed drug(s)**	
Yes	30(15.2)
No	167(84.8)
**Were you told the activities to avoid while taking this medicine(s)?**	
Yes	47(23.9)
No	150(76.1)
**Were you told if this medicine works well with other medicines?**	
Yes	53(27.0)
No	143(73.0)
**Were you told when to expect the medicine to work or how to know if it is working?**	
Yes	39(23.1)
No	130(76.9)
**Were you told to avoid certain foods/ medications?**	
Yes	61(30.5)
No	139(69.5)
**Were you told of any special storage condition for your medicine?**	
Yes	49(25.4)
No	144(74.6)
**AFTER CONSULTING THE DOCTOR**	n (%)
**Do you call your doctor when you have problems with the medicine?**	
Yes	31(15.8)
No	165( 84.2)
**Do you call your doctor/pharmacist if you think you have troublesome side effects?**	
Yes	39(19.8)
No	158(80.2)
**Do you know the date of your next clinic visit?**	
Yes	188(94.9)
No	10(5.1)

**Table 4:** Mean difference analysis for different steps before, during and after consultation with doctor

Variable	N	Steps before consultation Mean (SD)	95% Confidence interval	P-value	Steps during consultation Mean (SD)	95% confidence interval	P-value	Steps after consultation Mean (SD)	95% Confidence interval	P-value
**Age(years**)**^a^**										
˂30	13	4.38(2.18)	3.09-5.70	0.361	7.23(2.35)	5.81-8.65	0.076	1.69(1.11)	1.02-2.36	0.065
30-60	77	3.9(1.46)	3.62-4.29		7.26(2.62)	6.66-7.86		1.32(0.70)	1.17-1.48	
˃60	109	4.25(1.40)	3.98-4.51		6.55(1.75)	6.22-6.88		1.21(0.68)	1.08-1.34	
**Marital status^a^**										
Single	12	3.83(2.08)	2.51-5.16	*0.033	7.00(2.76)	5.24-8.76	0.792	1.67(1.23)	0.88-2.45	0.285
Married	151	4.03(1.49)	3.79-4.27		6.80(2.19)	6.45-7.15		1.26(0.70)	1.15-1.38	
Divorced	19	5.00(0.82)	4.61-5.39		7.26(2.21)	6.20-8.33		1.37(0.76)	1.00-1.74	
Widowed	6	3.50(0.55)	2.93-4.07		7.33(1.97)	5.27-9.40		1.50(.055)	0.93-2.07	
**Occupation**										
Government employed	36	3.94(1.37)	3.48-4.40	0.162	7.78(3.02)	6.75-8.80	*˂0.034	1.86(1.02)	1.52-2.21	*˂0.001
Corporate employed	34	3.91(1.50)	3.39-4.44		6.91(2.43)	6.06-7.76		1.12(0.59)	0.91-1.32	
Self employed	32	3.63(1.48)	3.09-4.16		6.56(1.39)	6.06-7.76		1.03(0.40)	0.89-1.18	
Unemployed	43	4.44(1.40)	4.01-4.87		6.30(1.73)	6.06-7.05		1.35(0.75)	1.12-1.58	
Retired	43	4.12(1.42)	3.68-4.55		6.74(1.62)	6.25-7.24		1.16(0.53)	1.00-1.33	
**Educational status^a^**										
Primary	61	3.57(1.54)	3.18-3.97	*˂0.001	6.25(1.26)	5.92-6.57	*˂0.001	1.02(0.50)	0.89-1.14	*˂0.001
Secondary	45	4.29(1.56)	3.82-4.76		7.36(2.13)	6.71-8.00		1.40(0.75)	1.17-1.163	
Tertiary	57	4.26(1.30)	3.92-4.61		7.60(2.87)	6.83-8.36		1.60(0.88)	1.36-1.83	
None	31	4.94(1.12)	4.52-5.35		6.10(1.76)	5.45-6.74		1.10(0.54)	0.90-1.29	

Test conducted: a = ANOVA, SD = Standard Deviation

* P< 0.05 is statistically significant.

**Table 5:** Association between educational status and steps taken to avoid medication errors before, during and after consultation with doctor

		Educational Status							
		Primary	Secondary	Tertiary	None	Total	χ^2^	df	P-Value
**Before Consultation**	**Inadequate Steps**	42	37	19	12	110	13.164	3	*<0.004
	**Good Steps**	19	20	26	19	84			
	**Total**	61	57	45	31	194			
									
**During Consultation**	**Inadequate Steps**	55	20	27	26	128	38.404	3	*<0.005
	**Good Steps**	6	25	30	5	66			
	**Total**	61	45	57	31	194			
									
**After Consultation**	**Inadequate Steps**	57	32	34	29	152	25.555	3	*<0.001
	**Good Steps**	4	13	23	2	42			
	**Total**	61	45	57	31	194			

Cramer’s V = 0.260 (before visit), 0.445 (during visit), 0.368 (after visit), P < 0.05 is statistically significant

**Table 6:** Association between occupational status and steps taken to avoid medication errors during and after consultation with doctor

		Occupation								
		Government Employed	Corporate Employed	Self Employed	Unemployed	Retired	Total	χ^2^	df	P-Value
**During Consultation**	**Inadequate Steps**	12	22	26	34	30	124	23.992	3	*˂0.001
	**Good Steps**	24	12	6	9	13	64			
	**Total**	36	34	32	43	43	188			
**After Consultation**	**Inadequate Steps**	17	30	31	31	35	144	28.226	4	*<0.005
	**Good Steps**	19	4	1	12	12	44			
	**Total**	36	34	32	43	47	188			

Cramer’s V = 0.357(during), 0.388 (after); P < 0.05 is statistically significant

## Discussion

In this study, we found that before clinic visits, most of the patients knew their doctor's names, and were not on any non-prescribed drugs. However, only a few had their doctor's phone number and seldom monitored their blood pressure. During consultation with their doctors, a little less than half of the patients knew the name of their prescribed drugs; majority were informed on how to take their drugs but only about a fifth were made aware of the side effects of their drugs or told how to deal with the problems that may arise from these side effects. Most of the patients agreed that it was not safe to take their drugs together with alcohol. Less than a third of the patients admitted that they were informed on the foods, medications and activities to avoid while on drugs, and how to know if the drug is working. After consultation with their doctors, few patients reported to the doctor or pharmacist about any troublesome side effects they experienced. Additionally, our findings suggest that patients who were government-employed and had tertiary education took the best steps in avoiding medication errors. However, few limitations of this study deserve consideration for better understanding and interpretations of the findings. First, the outcome of this study may not be generalized to the entire population of hypertensive patients in Nigeria considering that the study was conducted in a single healthcare facility even though it was a tertiary hospital. Secondly, patients' responses may vary if measured at different times as cross-sectional design was utilized. Finally, this study could only determine associations between dependent and independent variables rather than cause-effect relationship. Our results demonstrate that before consultation with their doctors, most of the patients knew the doctors' names. However, they did not have their contact phone numbers and their blood pressure was not regularly monitored at home. It could probably be that the physicians undermine the value of providing patients with such vital information as their phone numbers, the hospital policy does not permit such or the patients fail to make such requests from their doctors. Known hypertensive patients are expected to check their blood pressure regularly. This might not occur if they are not informed of the relevance or they do not have access to the monitoring device. The application of modern electronic devices such as mobile phones in medical care in the growing field of telemedicine cannot be overemphasized as it aids easy and prompt communication between the healthcare providers and the patients [18, 19]. Therefore, medication errors might be averted if patients are provided with names and cell phone numbers of their doctors (especially in chronic disease states, like hypertension), and are well informed on the need for routine blood pressure measurements.

During consultation with their doctors, our findings showed that although the doctors informed majority of the patients of their diagnosis, less than half of the patients were told the name(s) of their medicines. However, a study in Saudi Arabia reported that 70.5% of the patients assessed were informed of the name(s) of their medications [20], although a higher number (96.3%) was reported in a study in the United States [21]. Perhaps, the lower percentage of patients who were informed of the name(s) of their medications as reported in the current study may be because the physicians want to discourage irrational self-medication which is a common practice in Nigeria. Furthermore, most of the patients in the our study were given adequate directives on their medication use, which is similar to that reported in Saudi Arabia (75.9%) [20]. However, it was higher in the United States (99.1%) [21] and lower in a study in India (61.3%) [22]. Our findings may be as a result of doctors' awareness of the positive outcomes such as improved drug adherence, associated with patients' understanding of their medication use. Our study demonstrated that very few of the patients were informed of the possible side effects of their drugs. Similar findings were reported by Gad et al in Saudi Arabia (17%) [20]. Interestingly, a study in California, United States of America, found that a physician-targeted educational session enhanced patient ratings of physician communication about new drug prescriptions [21]. Another study in a North Indian city showed that although a considerable number of patients knew about the therapeutic effects of the drugs prescribed and their mode of administration, they lacked information regarding the side effects, warnings and information their doctors may require at the next consultation [22]. The lower awareness of side effects of medication in our study may be a strategy employed by the physicians to downplay the problems related to the drugs and increase patients' medication adherence. This may not be ideal as patients are more likely to discontinue a drug due to a side effect if they were not prior informed.

Also, in this study, barely one third of the patients were aware of the activities, food and medications to avoid while on their antihypertensive drugs, although most of them knew that it was not safe to take alcohol together with their medications. Indermitte et al [23] reported that half of the patients assessed in their study in Switzerland admitted being informed of these interactions. However, our finding was higher than the 15% reported in Saudi Arabia [20]. The explanation for this low percentage observed in this study could be because the doctors expected the pharmacists to counsel the patients on such issues as they come to the pharmacy unit for their medications. After consultation with their doctors, we found that only very few of the patients contact their doctors and/or pharmacists when they have problems with their medications or are experiencing troublesome side effects. Possible explanation for this finding could be that patients were not adequately informed on the appropriate course of action to take in such circumstances during clinic visits, or perhaps the patients who suffer from such troublesome medication side effects refrain from reporting the problem for fear of being seen as questioning the prescribers' clinical knowledge, skills and competency. Some physicians refrain from discussing potential side effects with their patients with the concern that such information might lead to an increase in the side effects experienced by these patients [24]. However, physicians are advised to inform patients of potential side effects prior to starting a new medication as this does not lead to an increased incidence of those side effects, and patients are encouraged to promptly report adverse experiences during medication use [24, 25]. In the present study, patients who had tertiary education and were government-employed took the best steps in avoiding medication errors before, during and after consulting their doctors. Our finding is consistent with the outcome of several studies which have shown a positive association between the knowledge of hypertension treatment and adherence with antihypertensive medications [25-27]. This observation probably could be because individuals with higher education appreciate the value of good health, easily comprehend and adhere to treatment instructions more than those with little or no formal education. Additionally, civil servants are more likely to have attained tertiary education, hence are better positioned to understand their drug treatment plan and avert any potential danger owing to medication use. For future studies, we suggest research for an in-depth understanding of how physicians and patients perceive medication errors, their meanings and implications to them.

## Conclusion

Patients that took good steps in avoiding medication errors before consulting with their doctors were likely to avoid errors in the consulting room. Also, patients that avoided medication errors at the consulting room were more likely to avoid medication errors thereafter. Those with tertiary education or the government-employed took the best steps in avoiding medication errors during and after consultation with their doctor. The involvement of patients in the management of their health care has been shown to reduce the incidence of medication errors in both inpatients and outpatients. Hence, health care professionals should as a matter of necessity provide patients with adequate information pertaining to their drug therapy. Additionally, patients should be encouraged to report to their health professionals, any observed medication-related problems.

### What is known about this topic

- Medication errors occur in chronic disease states, including hypertension;
- Medication errors could be minimized or prevented entirely if patients are actively involved in the management of their drug therapy.

### What this study adds

- Patients with higher level formal education are better at avoiding medication errors;
- Patients with chronic disease states need to be educated on self-management such as hypertensive patients regularly monitoring their blood pressure;
- Patients need to be educated about the need to inform their health professionals about any troublesome side effects from the use of their medications.

## Competing interests

The authors declare no competing interests.
